# Willingness to Adopt mHealth Among Chinese Parents During the COVID-19 Outbreak: Cross-sectional Questionnaire Study

**DOI:** 10.2196/23155

**Published:** 2021-01-27

**Authors:** Siyu Yang, Yijing Chen, Leshan Zhou, Yuting Huang, Jiahui Dai

**Affiliations:** 1 Nursing Department Tongji Hospital of Tongji Medical College Huazhong University of Science and Technology Wuhan China; 2 Wuhan Mental Health Center–affiliated Tongji Medical College Huazhong University of Science and Technology Wuhan China; 3 Xiangya Nursing School Central South University Changsha China; 4 Sydney School of Public Health Faculty of Medicine and Health University of Sydney Sydney Australia

**Keywords:** mHealth, parents, child health at home, COVID-19

## Abstract

**Background:**

Parental involvement in mobile health (mHealth) to consult with medical professionals appears to be prevalent in China with the rapid development of the internet. More parents with busy jobs have chosen to use mHealth. During the ongoing COVID-19 outbreak, mHealth can assist with health promotion, directions for medication use, and disease diagnosis via online chat and video consultation without contacting others. To our knowledge, no studies have been performed to explore the role of mHealth in parents’ attitudes toward child health care at home during the COVID-19 outbreak.

**Objective:**

This study aims to identify the associated factors of willingness to adopt mHealth among Chinese parents during the COVID-19 outbreak and to explore the correlation between the frequency of adopting mHealth and parents’ attitudes toward child health care at home.

**Methods:**

Chinese parents were asked to complete an online survey from January 25 to February 15, 2020. The questionnaire comprised of two parts with a total of 16 items, including parents’ demographic variables and attitudes toward child health care at home. By multivariate logistic regression, we explored factors associated with parents’ willingness to adopt mHealth during the COVID-19 outbreak. Pearson chi-square tests were used to reveal the correlation between the frequency of adopting mHealth and parents’ attitudes toward child health care at home.

**Results:**

A total of 254 parents enrolled, and 202 (79.5%) parents were willing to adopt mHealth during the COVID-19 outbreak. Parents’ age (26-35 years: adjusted odds ratio [AOR] 8.114, 95% CI 1.471-44.764), parents’ interest in the COVID-19 pandemic (moderate: AOR 8.753, 95% CI 2.009-38.127; high: AOR 22.194, 95% CI 5.509-89.411), the source that recommended mHealth (medical health providers: AOR 4.257, 95% CI 1.439-12.596), the presence of chronic disease in their children (yes: AOR 20.844, 95% CI 4.600-94.443), parents’ duration of daily internet use (4-6 hours: AOR 6.487, 95% CI 1.870-22.495; >6 hours: AOR 8.766, 95% CI 1.883-40.804), and adoption of mHealth before the COVID-19 outbreak (yes: AOR 3.413, 95% CI 1.234-9.444) were significantly correlated with the parents’ willingness to adopt mHealth during the COVID-19 outbreak. The frequency of mHealth use among parents was correlated with their behaviors in regard to handwashing (χ^2^_6_=18.967, *P=.*004), mask wearing (χ^2^_6_=45.364, *P*<.001), frequency of leaving the home (χ^2^_6_=16.767, *P=*.01), room disinfection and ventilation (χ^2^_6_=19.515, *P=*.003), temperature checking (χ^2^_6_=17.47, *P=.*007), and mental health care of children (χ^2^_6_=63.810, *P*<.001) during the COVID-19 pandemic.

**Conclusions:**

We found various objective factors that were associated with parents’ willingness to adopt mHealth during the COVID-19 outbreak. Overall, parents’ willingness to adopt mHealth was high. The frequency of mHealth use among parents was correlated with their attitudes toward child health care at home. The option of mHealth to patients at home during the COVID-19 outbreak would be beneficial for education and improvement in self-management of child health care at home.

## Introduction

### Background

COVID-19 has caused an ongoing pandemic and is an important public health concern. The major transmission modes of COVID-19 are airborne droplets from coughing or sneezing and direct contact with contaminated surfaces such as doorknobs, dishes, and handrails [1]. Particular circumstances including tracheal incubation or opening suction in a hospital, staying in confined spaces with infected people, and fomites attaching to ventilation systems can result in mass infection [1].

Infants and children are a typically vulnerable population due to immaturity of the respiratory tract and hypoimmunity [2]. According to an analysis of 2143 pediatric cases in China, the median age of children who are infected is 7 years [3]. The majority of severe pediatric cases arose from exposure to infected family members, and few were infected in a hospital or as a result of travel [4]. To effectively prevent and control the COVID-19 outbreak among Chinese children, the National Health Commission issued guidelines termed “Epidemiological characteristics and prevention and control measures of Corona Virus Disease 2019 in children,” which clearly state that children are required to be isolated at home under the parents’ supervision [5]. In addition, experts also suggest that parents do not take children to the hospital to avoid cross infection [6]. To prevent large scale gatherings, all regional governments of China shut down schools and colleges, and decreased the run time of public transportation. Residents dwelling in high-risk areas are forbidden to go out except to acquire daily necessities and to visit the hospital [6].

In China, more than 1.3 billion people access the internet via their mobile phones, which has become an indispensable part of daily life [7]. The application of mobile health (mHealth) is widely recommended for Chinese parents to replace visiting a hospital during the COVID-19 outbreak. mHealth is defined as the use of wireless electronic devices to transmit various contents and medical services among patients and caregivers. Besides routine use, Chinese people could use mHealth during the COVID-19 pandemic through mobile phone apps [8,9], the hospital website, and doctors’ official social media accounts on WeChat or Alipay to get primary diagnoses between common cold or flu and pneumonia, achieve self-monitoring, access online lectures about COVID-19 prevention, and purchase essential medicine from online diagnosis. Specific individuals could also acquire urgent care in emergency situations by using mHealth to contact specialists or hospitals designed for delivery, patients with chronic disease, or terminal cancer. mHealth is viewed as an easily accessible, cost-efficient approach to enhancing adherence to medication, expanding access to medical care, and increasing the number of medical consultations [10]. Overall, mHealth has proven to be a great success for the management of chronic disease [11], remote monitoring of weight control [12], improvement of child vaccine coverage, and neonatal care among young mothers [13].

### Objectives

Though robust evidence highlights the potential benefits of mHealth, individual willingness to adopt mHealth is a decisive factor. Therefore, the purpose of this paper is to explore factors associated with willingness to adopt mHealth among Chinese parents during the COVID-19 outbreak for better promotion of mHealth in China and to investigate the correlation between frequency of adopting mHealth during the COVID-19 outbreak in 1 month and parents’ attitudes toward child health at home.

## Methods

### Definition of Variables

The willingness to adopt mHealth among Chinese parents was measured with a yes or no question.

mHealth in China is defined as the dissemination of medical information, consultation about disease diagnosis and treatment, postoperative care management, mental health care, and making medical appointments via mobile phone apps or social media [14].

### Participant Recruitment

This cross-sectional study was conducted between January 25, 2020, and February 15, 2020. Data were collected using structured questionnaires based on a literature review. A total of 12 participants were recruited to test the original questionnaire and provide feedback, ensuring the questionnaire was understandable. Regarding the COVID-19 pandemic, only an online version of this questionnaire was used for distributing the survey. The online questionnaire link was forwarded to social media groups, such as breastfeeding groups and parental involvement in kindergarten or primary school online groups, and posted on pediatric researchers’ social media webpages, where health education about children is given, to maximize recruitment of respondents. If parents were interested in our study, they could visit our questionnaire through a link, and a description of this study was shown on the first page of the online questionnaire. Parents could only access the questionnaire by clicking “agree” after reading the consent information on the first page, and the submission was accepted when all items were completed.

Parents who met any of the following criteria were excluded: completed the questionnaire in less than 120 seconds, their child was older than 14 years, the parent has not lived with their child during the COVID-19 pandemic, one or both parents have a confirmed case of COVID-19, parent has no internet access, more than one questionnaire was submitted from the same Internet Protocol address, and the questionnaire had missing items. Two researchers, who were master’s degree students, selected the valid questionnaires in accordance with the exclusion criteria. This study was approved by the Ethics Committees of Xiangya Nursing School, Central South University. In total, 18 questionnaires were considered substandard and excluded, and 254 Chinese parents participated in the online survey with an effective rate of 93.38% (254/272).

### Measurement of Variables

Following a literature review by our research team, consisting of one professor and five graduate students, a structured questionnaire was designed for this study. The 23-item questionnaire was comprised of two parts: (1) parents’ demographic variables including gender, age, education level, family annual income, occupation, age of the child, residence, attention to the pandemic of COVID-19, how they heard about mHealth, presence of chronic disease in their child, their use of mHealth before the COVID-19 outbreak, confirmed or suspected case of COVID-19 in their community, and duration of daily internet use, and (2) parents attitudes toward child health at home in regard to diet, exercise, personal hygiene, sleep quality, and mental care. In total, 30 parents were involved in the pilot study to modify the statements in the questionnaire. Subsequently, “worries about privacy disclosure” and “the frequency of nutrition supplement intake among children” were deleted. Most parents reported that their name or phone number is hidden when using mHealth, and other confounding factors such as residence, family income, and education level were related to the intake of nutritional supplements.

### Statistical Analysis

Each questionnaire was screened by two separate researchers and inputted into SPSS.V.22 (IBM Corp) for analysis. Means and SDs were used to describe the continuous variables with normal distribution. Numbers and percentages were used to represent categorical variables. The associated factors of willingness to adopt mHealth among Chinese parents during the COVID-19 outbreak were analyzed by binary and multivariate logistic regression. To eliminate the effects of confounding variables on the results, only variables with a *P* value <.20 following bivariate logistic regression analyses were entered into the logistic regression. Correlation chi-square tests were used to determine the correlation between the frequency of adopting mHealth during the COVID-19 outbreak in 1 month and parents’ attitudes toward child health at home. Corrected *P* values <.05 were considered statistically significant.

## Results

### Sociodemographic Characteristics

In total, 254 parents were recruited, and 172 (67.7%) were female. The age ranged from 26 to 35 years. Overall, 175 (68.9%) parents had a bachelor’s degree or above. Almost half of the parents’ family annual income reached more than US $7700. There were 179 (70.5%) parents who had jobs working in information technology, medicine, service, or other industries, and others were self-employed or jobless. In total, 165 (64.9%) parents were living in urban areas during the COVID-19 outbreak. All details about the parents’ sociodemographic characteristics are presented in Table 1.

**Table 1:** Demographics of parents (N=254).

Variables		Willingness to adopt mHealth^a^	
		No, n (%)	Yes, n (%)
**Gender**			
	Male	20 (24.4)	62 (75.6)
	Female	32 (18.6)	140 (81.4)
**Age (years)**			
	18-25	6 (37.5)	10 (62.5)
	26-35	37 (18.7)	161 (81.3)
	≥36	9 (22.5)	31 (77.5)
**Education level**			
	Middle school or below	12 (20)	48 (80)
	High school	6 (31.6)	13 (68.4)
	University or college	29 (20.1)	115 (79.9)
	Master’s degree or above	5 (16.1)	26 (83.9)
**Family annual income (US $)**			
	<1600	10 (18.5)	44 (81.5)
	1600-7700	15 (20.3)	59 (79.7)
	7700-16,000	14 (29.2)	34 (70.8)
	>16,000	13 (16.7)	65 (83.3)
**Occupation**			
	Medical care	4 (14.3)	24 (85.7)
	IT^b^	2 (14.3)	12 (85.7)
	Service	14 (18.2)	63 (81.8)
	Other	19 (31.7)	41 (68.3)
	Self-employed	6 (12.5)	42 (87.5)
	Jobless	7 (5.5)	20 (74.1)
**Age of the child (years)^c^**			
	<3	33 (18.8)	143 (81.3)
	3-6	16 (23.9)	51 (76.1)
	7-14	3 (27.3)	8 (72.7)
**Residence**			
	Urban	21 (23.6)	68 (76.4)
	Rural	31 (18.8)	134 (81.2)
**Attention to the COVID-19 pandemic**			
	Low	13 (59.1)	9 (40.9)
	Moderate	14 (26.4)	39 (73.6)
	High	25 (14)	154 (86)
**The recommendation about mHealth received from**			
	Media (phone message, internet, TV program)	19 (32.3)	40 (67.8)
	Community or people you are familiar with	19 (25.7)	55 (74.3)
	Medical health providers	14 (11.6)	107 (88.4)
**Presence of chronic disease in children**			
	Yes	3 (3.8)	75 (96.2)
	No	49 (27.8)	127 (72.2)
**Adoption of mHealth before the COVID-19 outbreak**			
	Yes	7 (9.9)	64 (90.1)
	No	45 (24.6)	138 (75.4)
**Confirmed or suspected case was found in your community**			
	Yes	9 (16.4)	46 (83.6)
	No	43 (21.6)	156 (78.4)
**Duration of daily internet use (hours)**			
	<2	12 (54.5)	10 (45.5)
	2-4	19 (32.8)	39 (67.2)
	4-6	16 (12.9)	108 (87.1)
	>6	5 (10)	45 (90)

^a^mHealth: mobile health.

^b^IT: information technology.

^c^Respondents with more than one child were asked to provide the age of their youngest child.

### Willingness to Adopt mHealth

The majority of the 254 parents (n=202, 79.5%) reported that they were willing to adopt mHealth during the COVID-19 outbreak (Table 1). The proportion of parents with a high or moderate interest in the COVID-19 pandemic who were willing to adopt mHealth was higher than those with low interest (39/53, 73.6% and 154/179, 86% vs 9/22, 40.9%, respectively). Willingness to adopt mHealth was highest among parents of children with chronic diseases (75/78, 96.2%). Willingness to adopt mHealth increased with parents’ duration of daily internet use.

### Factors Associated With Willingness to Adopt mHealth

Results from the bivariate analyses demonstrated that age, interest in the pandemic, the source that recommended mHealth, the presence of a chronic disease in children, duration of daily internet use, and use of mHealth before the COVID-19 outbreak were associated with willingness to adopt mHealth during the pandemic. The multivariate logistics regression model indicated that the parents’ age (26-35 years: adjusted odds ratio [AOR] 8.114, 95% CI 1.471-44.764), parents’ interest in the COVID-19 pandemic (moderate: AOR 8.753, 95% CI 2.009-38.127; high: AOR 22.194, 95% CI 5.509-89.411), the source that recommended mHealth (medical health providers: AOR 4.257, 95% CI 1.439-12.596), presence of chronic disease in children (yes: AOR 20.844, 95% CI 4.600-94.443), parents’ duration of daily internet use (4-6 hours: AOR 6.487, 95% CI 1.870-22.495; >6 hours: AOR 8.766, 95% CI 1.883-40.804), and adoption of mHealth before the COVID-19 outbreak (yes: AOR 3.413, 95% CI 1.234-9.444) were significantly correlated with the parents’ willingness to adopt mHealth during the COVID-19 outbreak (Table 2).

The odds of being willing to adopt mHealth were 8.1 times greater in parents aged from 26 to 35 years than parents aged from 18 to 25 years (*P*=.02). The odds of being willing to adopt mHealth were 8.6 times greater in respondents with moderate interest in the pandemic than respondents with low interest (*P*=.004). Meanwhile, the odds of being willing to adopt mHealth were 22.2 times greater in participants with high interest in the pandemic than respondents with low interest in the pandemic (*P*<.001). The odds of being willing to adopt mHealth were 4.3 times greater in parents who were recommended to use mHealth by a medical health provider than parents who received the recommendation from the media (*P=*.009). The odds of being willing to adopt mHealth were 21 times greater in parents of children with chronic diseases than parents of children without chronic diseases (*P*<.001). The odds of being willing to adopt mHealth were 6.5 times greater in respondents with 4-6 hours of daily internet use than respondents with 2 hours of daily internet use (*P=*.003). Moreover, the odds of being willing to adopt mHealth were 8.8 times greater in participants with >6 hours of daily internet use than respondents with 2 hours of daily internet use (*P*=.006). Furthermore, odds of being willing to adopt mHealth were 3.4 times greater in parents who had ever adopted mHealth than those who had not (*P*=.02).

**Table 2:** Multivariate analyses of factors associated with willingness to adopt mHealth among Chinese parents (N=254).

Variable		Willingness to adopt mHealth^a^, n (%)		Crude OR^b^ (95% CI)	Adjusted OR (95% CI)	*P* value
		No	Yes			
**Age (years)**						
	18-25	6 (37.5)	10 (62.5)	1	1	N/A^c^
	26-35	37 (18.7)	161 (81.3)	11.591 (1.805-74.448)	8.114 (1.471-44.764)	.02
	≥36	9 (22.5)	31 (77.5)	12.513 (1.362-114.971)	5.794 (0.841-39.913)	.07
**Attention to the COVID-19 pandemic**						
	Low	13 (59.1)	9 (40.9)	1	1	N/A
	Moderate	14 (26.4)	39 (73.6)	13.113 (2.119-84.124)	8.753 (2.009-38.127)	.004
	High	25 (14)	154 (86)	31.889 (6.395-159.020)	22.194 (5.509-89.411)	<.001
**The recommendation about mHealth received from**						
	Media (phone message, internet, TV program)	19 (24.4)	59 (75.6)	1	1	N/A
	Medical health providers	14 (14.3)	84 (85.7)	4.710 (1.382-16.049)	4.257 (1.439-12.596)	.009
**Presence of chronic disease in children**						
	No	3 (3.8)	75 (96.2)	1	1	N/A
	Yes	49 (27.8)	127 (72.2)	30.571 (5.552-168.331)	20.844 (4.600-94.443)	<.001
**Duration of daily internet use (hours)**						
	<2	12 (54.5)	10 (45.5)	1	1	N/A
	4-6	16 (12.9)	108 (87.1)	6.860 (1.591-29.575)	6.487 (1.870-22.495)	.003
	>6	5 (10)	45 (90)	6.794 (1.141-40.455)	8.766 (1.883-40.804)	.006
**Use of mHealth before the COVID-19 outbreak**						
	No	45 (24.6)	138 (75.4)	1	1	N/A
	Yes	7 (9.9)	64 (90.1)	3.759 (1.185-11.928)	3.413 (1.234-9.444)	.02

^a^mHealth: mobile health.

^b^OR: odds ratio.

^c^N/A: not applicable.

### Correlation Between Frequency of Using mHealth During the COVID-19 Outbreak in 1 Month and Parents’ Attitudes Toward Child Health at Home

Table 3 presents the results of the correlation between frequency of using mHealth during the COVID-19 outbreak in 1 month and parents’ attitudes toward child health at home. Frequency of using mHealth during the COVID-19 outbreak in 1 month was associated with parents’ attitudes toward ventilation and daily disinfection of their child’s room (*P*=.003) and guidance for the child on washing hands properly every time (*P*=.004). Specific actions to prevent children from contracting COVID-19, such as instructing them to wear medical masks appropriately (*P*<.001) and reducing the frequency of children leaving the home (*P*=.01), were correlated with the frequency of using mHealth during the COVID-19 outbreak in 1 month. In addition, the frequency of using mHealth during the COVID-19 outbreak in 1 month was significantly correlated with parents’ attitudes toward checking children’s temperature regularly (*P*=.007) and ensuring mental health care at home (*P*<.001).

**Table 3:** Correlation between parents’ attitudes toward child health at home and the frequency of adopting mHealth in 1 month (N=254).

Item		Frequency of mHealth^a^ service use, n (%)				Chi-square (*df*)	*P* value
		0	1–2	3–4	>4		
**Reduce the frequency of children leaving the home**						16.767 (6)	.01
	Never/seldom	10 (26.6)	7 (23.5)	8 (16.7)	9 (19)		
	Sometimes	31 (21.5)	4 (21.6)	17(33.3)	17 (22.4)		
	Often/always	38 (51.9)	40 (54.9)	41(50)	32 (58.6)		
**Ventilation and daily disinfection of child’s room**						19.515 (6)	.003
	Never/seldom	13(16.5)	4 (7.8)	2 (3)	10 (17.2)		
	Sometimes	3 (3.8)	9 (17.6)	6 (9.1)	11 (19)		
	Often/always	63 (79.7)	38 (74.5)	58 (87.9)	37 (63.8)		
**Guidance for the child on washing hands properly every time**						18.967 (6)	.004
	Never/seldom	22 (27.8)	9 (17.6)	11 (16.7)	6 (10.3)		
	Sometimes	24 (30.4)	14 (27.5)	25 (37.9)	9 (15.5)		
	Often/always	33 (41.8)	28 (54.9)	30 (45.5)	43 (74.1)		
**Instructing children to wear medical masks appropriately**						45.364 (6)	<.001
	Never/seldom	11 (13.9)	13 (25.5)	13 (19.7)	3 (5.2)		
	Sometimes	45 (57)	27 (52.9)	28 (42.4)	11 (19)		
	Often/always	23(29.1)	11 (21.6)	25 (25.2)	44 (75.9)		
**Cooking nutritional meals**						4.174 (6)	.65
	Never/seldom	20 (25.3)	16 (31.4)	16 (24.2)	15 (25.9)		
	Sometimes	28 (31.1)	18 (35.3)	32 (48.5)	22 (37.9)		
	Often/always	31 (27.1)	17 (33.3)	18 (27.3)	21 (36.2)		
**Improving child’s sleep quality**						6.996 (6)	.32
	Never/seldom	19 (24.1)	13 (25.5)	17 (25.8)	20 (34.5)		
	Sometimes	27 (34.2)	21 (43.1)	23 (34.8)	25 (43.1)		
	Often/always	33 (27.4)	16 (31.4)	26 (39.4)	13 (22.4)		
**Encouraging child to exercise at home**						6.188 (6)	.40
	Never/seldom	11 (13.9)	10 (19.6)	10 (15.2)	6 (10.3)		
	Sometimes	17 (21.5)	12 (23.5)	23 (34.8)	14 (24.1)		
	Often/always	51 (64.6)	29 (56.9)	33 (50)	38 (65.5)		
**Relieving child’s negative emotions at home**						63.810 (6)	<.001
	Never/seldom	41 (51.9)	20 (39.2)	8 (12.1)	6 (10.3)		
	Sometimes	33 (41.8)	30 (58.8)	32 (48.5)	34 (58.6)		
	Often/always	5 (6.3)	1 (2)	26 (39.4)	18 (31)		
**Checking child’s temperature regularly**						17.847 (6)	.007
	Never/seldom	32 (40.5)	10 (19.6)	15 (22.7)	8 (13.8)		
	Sometimes	20 (25.3)	10 (19.6)	16 (24.2)	17 (29.3)		
	Often/always	27 (34.2)	31 (60.8)	35 (53)	33 (56.9)		
**Update knowledge about COVID-19 prevention**						5.041 (6)	.53
	Never/seldom	21 (26.6)	12 (23.5)	11 (16.7)	11 (19)		
	Sometimes	17 (21.5)	11 (21.6)	22 (33.3)	13(22.4)		
	Often/always	41 (51.9)	28 (54.9)	33 (50)	34 (58.6)		

^a^mHealth: mobile health.

## Discussion

### Principal Findings

Our primary finding was that the parents’ age and interest in the COVID-19 pandemic, the source that recommended mHealth, the presence of a chronic disease in their children, duration of daily internet use among parents, and adoption of mHealth service before the COVID-19 outbreak were significantly correlated with parents’ willingness to adopt mHealth during the COVID-19 outbreak. In addition, the frequency of mHealth use for parents was correlated with their attitudes toward handwashing, mask wearing, frequency of going out, room disinfection and ventilation, temperature checking, and care of children’s mental health during the COVID-19 pandemic.

The findings show that, overall, parents’ willingness to adopt mHealth during the COVID-19 pandemic was high (202/254, 79.5%), which is supported by another study that reported willingness to adopt mHealth at 80% [15]. However, another study conducted in China reported that just 66.1% (725/1097) of participants were willing to participate in mHealth programs for patients with chronic diseases [7]. James and Harville [16] showed an almost identical result (n=881, 67%). One possible explanation is that the proportion of participants who were willing to engage with various components of mHealth technology varied from 59% to 81% [17].

Our study reveals several factors influencing parents’ willingness to adopt mHealth, including age, interest in the COVID-19 pandemic, the source that recommended mHealth, the presence of a chronic disease in their children, duration of daily internet use, and adoption of mHealth before the COVID-19 outbreak. Age was shown to be significantly related to the parents’ willingness to adopt mHealth in our study, which is in line with previous published studies on mHealth acceptance factors, where age was an important factor among both patients and medical professionals [18-20]. Importantly, age has specific moderating effects on the adoption of mHealth [18]. However, age in our study did not play as significant a role as in other studies [20], and one possible explanation for this may be the varied age group included [21].

We found that the odds of willing to adopt mHealth were greater in parents with higher levels of attention to the COVID‑19 pandemic than those with lower attention, which is consistent with previous studies on the adoption of eHealth services during the COVID-19 pandemic [22,23]. Previous studies have confirmed a direct relationship between perception risk and technology adoption [24,25]. The outbreak of COVID-19 is regarded as a facilitating factor for the adoption and acceptance of technology [26]. Many measures were taken to accelerate the adoption of mHealth, including practical guidance for individual practices to quickly adopt mHealth in response to COVID-19 [22,27]. Moreover, the range of providers who could deliver care through mHealth was broadened, and rules around patient eligibility and audiovisual equipment requirements were relaxed specifically to address COVID-19 [22,28].

This study demonstrates that the source of referral is associated with parents’ willingness to participate in mHealth programs, specifically the odds were greater in patients who were recommended mHealth by medical professionals (98/254, 85.7%), which is consistent with previous findings that reported about one-third of patients would likely be able to contact their doctors using an electronic device [20]. On the contrary, another study found that the source of referral for mHealth was through various media sources such as email. One possible reason is that all the users included were children with a median age of 6 years [29].

This study indicates that duration of daily internet use is associated with a willingness to adopt mHealth among parents, which is in line with a previous study that reported time spent on the internet is greatly associated with the level of eHealth literacy [30,31]. However, time spent on the internet was reported to be a nonsignificant factor in other studies; therefore, the small sample size and methodological differences may have played an important role in it [32,33].

Overall, our study found that the odds of willing to adopt mHealth was greater in parents of children with chronic diseases (>90%). This finding is consistent with a previous study that found the most patients who are chronically ill (>80%) would be willing to participate in mHealth programs in transitional countries [15]. Prior findings indicated that sharing personal health information and receiving support through social networking can benefit adolescents with a chronic illness [34-36]. The positive association is likely due to the patient’s improved awareness of the importance of mobile phones for care of chronic health conditions [37-40].

Previous use of mHealth was found to be greatly associated with the participants’ willingness to adopt mHealth in this trial. This finding is consistent with a previous study where respondents who had used WeChat before were more willing to adopt mHealth as a result of their greater familiarity and confidence in new technology [7]. One potential reason is that prior use experiences influence various beliefs and, consequently, willingness to use technology in a consumer context [41].

The frequency of mHealth use for parents was associated with their attitudes toward handwashing, mask wearing, frequency of going out, room disinfection and ventilation, temperature checking, and care of children’s mental health during the COVID-19 pandemic. A previous published study demonstrated the effectiveness of hand hygiene, mask wearing, and social distancing for the prevention of COVID-19 as well as other respiratory infectious diseases [42]. Interestingly, mask wearing and handwashing among children were found to be influenced by frequency of leaving the house, mother’s educational background, and father’s occupation [43]. Furthermore, mental health status was found to be a big issue during the crisis for children who were isolated and quarantined [44]. An increase in sedentary behavior was observed due to the pandemic, and students were found to be more depressed and anxious during this time [45]. Thus, psychological crisis interventions targeted to different pediatric age groups could be conducted to minimize the psychological trauma and subsequent psychosocial problems caused by the COVID-19 pandemic [46]. mHealth is an ideal tool for the control of communicable diseases. Social distancing has a significant role in cutting the transmission of the virus and decreasing the chance of face-to-face contact. Especially during the COVID-19 outbreak, mHealth could provide some recommendations about health management for people quarantined at home.

### Limitations

First, the sample size was insufficient. A study with a larger sample is recommended to further improve the representativeness of the study results. Second, social desirability bias and recall bias may have arose from self-reporting. Confounding or unknown factors omitted from the survey may also have caused residual confounding, and instrumental variable analysis should be used to control these confounding factors. Third, a cause-effect relationship could not be established due to the inherent nature of the cross-sectional study design.

### Conclusion

The COVID-19 pandemic has enormously changed health care systems worldwide, and internet-based medical care is likely to play a major role during the COVID-19 pandemic to increase widespread access to effective care and overcome the challenges and restrictions imposed by the outbreak [47]. We found various objective factors associated with parents’ willingness to adopt mHealth during the COVID-19 outbreak, and the frequency of mHealth use among parents was correlated with their attitudes toward child health at home. Furthermore, our study provides new insight into how parents cope with pandemic-related mental health problems in children. These findings provide valuable information for mHealth service providers and policy makers to develop policy and strategies for the successful implementation and acceleration of this technology’s adoption.

## Abbreviations

AOR: adjusted odds ratio
mHealth: mobile health

## References

[1] Sankar J, Dhochak N, Kabra SK, Lodha R (2020). COVID-19 in children: clinical approach and management. Indian J Pediatr.

[2] Kelvin AA, Halperin S (2020). COVID-19 in children: the link in the transmission chain. Lancet Infect Dis.

[3] Lu X, Zhang L, Du H, Zhang J, Li YY, Qu J, Zhang W, Wang Y, Bao S, Li Y, Wu C, Liu H, Liu D, Shao J, Peng X, Yang Y, Liu Z, Xiang Y, Zhang F, Silva RM, Pinkerton KE, Shen K, Xiao H, Xu S, Wong GWK, Chinese Pediatric Novel Coronavirus Study Team (2020). SARS-CoV-2 infection in children. N Engl J Med.

[4] To T, Viegi G, Cruz A, Taborda-Barata L, Asher I, Behera D, Bennoor K, Boulet L, Bousquet J, Camargos P, Conceiçao C, Gonzalez Diaz S, El-Sony A, Erhola M, Gaga M, Halpin D, Harding L, Maghlakelidze T, Masjedi MR, Mohammad Y, Nunes E, Pigearias B, Sooronbaev T, Stelmach R, Tsiligianni I, Tuyet Lan LT, Valiulis A, Wang C, Williams S, Yorgancioglu A (2020). A global respiratory perspective on the COVID-19 pandemic: commentary and action proposals. Eur Respir J.

[5] She J, Liu L, Liu W (2020). COVID-19 epidemic: disease characteristics in children. J Med Virol.

[6] (2020). Diagnosis and treatment protocol for novel coronavirus pneumonia (trial version 7). Chin Med J (Engl).

[7] Dai M, Xu J, Lin J, Wang Z, Huang W, Huang J (2017). Willingness to use mobile health in glaucoma patients. Telemed J E Health.

[8] Olson CA, McSwain SD, Curfman AL, Chuo J (2018). The current pediatric telehealth landscape. Pediatrics.

[9] Shigekawa E, Fix M, Corbett G, Roby DH, Coffman J (2018). The current state of telehealth evidence: a rapid review. Health Aff (Millwood).

[10] Qian W, Lam TT, Lam HHW, Li C, Cheung YT (2019). Telehealth interventions for improving self-management in patients with hemophilia: scoping review of clinical studies. J Med Internet Res.

[11] Cajita MI, Hodgson NA, Budhathoki C, Han H (2017). Intention to use mHealth in older adults with heart failure. J Cardiovasc Nurs.

[12] James DCS, Harville C (2018). Smartphone usage, social media engagement, and willingness to participate in mHealth weight management research among African American women. Health Educ Behav.

[13] Ruton H, Musabyimana A, Gaju E, Berhe A, Grépin KA, Ngenzi J, Nzabonimana E, Law MR (2018). The impact of an mHealth monitoring system on health care utilization by mothers and children: an evaluation using routine health information in Rwanda. Health Policy Plan.

[14] Liu S, Yang L, Zhang C, Xiang Y, Liu Z, Hu S, Zhang B (2020). Online mental health services in China during the COVID-19 outbreak. Lancet Psychiatry.

[15] Piette JD, Mendoza-Avelares MO, Milton EC, Lange I, Fajardo R (2010). Access to mobile communication technology and willingness to participate in automated telemedicine calls among chronically ill patients in Honduras. Telemed J E Health.

[16] James DCS, Harville C (2016). eHealth literacy, online help-seeking behavior, and willingness to participate in mHealth chronic disease research among African Americans, Florida, 2014-2015. Prev Chronic Dis.

[17] Abelson JS, Symer M, Peters A, Charlson M, Yeo H (2017). Mobile health apps and recovery after surgery: what are patients willing to do?. Am J Surg.

[18] Zhao Y, Ni Q, Zhou R (2018). What factors influence the mobile health service adoption? A meta-analysis and the moderating role of age. Int J Inf Manage.

[19] Chib A, van Velthoven MH, Car J (2015). mHealth adoption in low-resource environments: a review of the use of mobile healthcare in developing countries. J Health Commun.

[20] Illiger K, Hupka M, von Jan U, Wichelhaus D, Albrecht U (2014). Mobile technologies: expectancy, usage, and acceptance of clinical staff and patients at a university medical center. JMIR mHealth uHealth.

[21] Garavand A, Samadbeik M, Nadri H, Rahimi B, Asadi H (2019). Effective factors in adoption of mobile health applications between medical sciences students using the UTAUT model. Methods Inf Med.

[22] Scott BK, Miller GT, Fonda SJ, Yeaw RE, Gaudaen JC, Pavliscsak HH, Quinn MT, Pamplin JC (2020). Advanced digital health technologies for COVID-19 and future emergencies. Telemed J E Health.

[23] Hollander JE, Carr BG (2020). Virtually perfect? Telemedicine for Covid-19. N Engl J Med.

[24] Quaosar GMAA, Hoque MR, Bao Y (2018). Investigating factors affecting elderly's intention to use m-Health services: an empirical study. Telemed J E Health.

[25] Phichitchaisopa N, Naenna T (2013). Factors affecting the adoption of healthcare information technology. EXCLI J.

[26] Ting DSW, Carin L, Dzau V, Wong TY (2020). Digital technology and COVID-19. Nat Med.

[27] Mann DM, Chen J, Chunara R, Testa PA, Nov O (2020). COVID-19 transforms health care through telemedicine: evidence from the field. J Am Med Inform Assoc.

[28] Kichloo A, Albosta M, Dettloff K, Wani F, El-Amir Z, Singh J, Aljadah M, Chakinala RC, Kanugula AK, Solanki S, Chugh S (2020). Telemedicine, the current COVID-19 pandemic and the future: a narrative review and perspectives moving forward in the USA. Fam Med Community Health.

[29] Fiks AG, Fleisher L, Berrigan L, Sykes E, Mayne SL, Gruver R, Halkyard K, Jew OS, FitzGerald P, Winston F, McMahon P (2018). Usability, acceptability, and impact of a pediatric teledermatology mobile health application. Telemed J E Health.

[30] Mitsutake S, Shibata A, Ishii K, Oka K (2012). Association of eHealth literacy with colorectal cancer knowledge and screening practice among internet users in Japan. J Med Internet Res.

[31] van der Vaart R, van Deursen AJ, Drossaert CH, Taal E, van Dijk JA, van de Laar MA (2011). Does the eHealth Literacy Scale (eHEALS) measure what it intends to measure? Validation of a Dutch version of the eHEALS in two adult populations. J Med Internet Res.

[32] Tubaishat A, Habiballah L (2016). eHealth literacy among undergraduate nursing students. Nurse Educ Today.

[33] Robb M, Shellenbarger T (2014). Influential factors and perceptions of eHealth literacy among undergraduate college students. On-Line J Nurs Inform.

[34] Hether HJ, Murphy ST, Valente TW (2014). It's better to give than to receive: the role of social support, trust, and participation on health-related social networking sites. J Health Commun.

[35] Nicholas DB, Fellner KD, Frank M, Small M, Hetherington R, Slater R, Daneman D (2012). Evaluation of an online education and support intervention for adolescents with diabetes. Soc Work Health Care.

[36] Vaala S, Lee J, Hood K, Mulvaney S (2018). Sharing and helping: predictors of adolescents' willingness to share diabetes personal health information with peers. J Am Med Inform Assoc.

[37] Shibuta T, Waki K, Tomizawa N, Igarashi A, Yamamoto-Mitani N, Yamaguchi S, Fujita H, Kimura S, Fujiu K, Waki H, Izumida Y, Sasako T, Kobayashi M, Suzuki R, Yamauchi T, Kadowaki T, Ohe K (2017). Willingness of patients with diabetes to use an ICT-based self-management tool: a cross-sectional study. BMJ Open Diabetes Res Care.

[38] Mathieson K, Leafman JS, Horton MB (2017). Access to digital communication technology and perceptions of telemedicine for patient education among American Indian patients with diabetes. J Health Care Poor Underserved.

[39] Nijland N, van Gemert-Pijnen JEWC, Kelders SM, Brandenburg BJ, Seydel ER (2011). Factors influencing the use of a Web-based application for supporting the self-care of patients with type 2 diabetes: a longitudinal study. J Med Internet Res.

[40] Jemere AT, Yeneneh YE, Tilahun B, Fritz F, Alemu S, Kebede M (2019). Access to mobile phone and willingness to receive mHealth services among patients with diabetes in Northwest Ethiopia: a cross-sectional study. BMJ Open.

[41] Ajzen I, Fishbein M, Albarracín D, Johnson BT, Zanna MP (2005). The influence of attitudes on behavior. The Handbook of Attitudes.

[42] Chiu N, Chi H, Tai Y, Peng C, Tseng C, Chen C, Tan BF, Lin C (2020). Impact of wearing masks, hand hygiene, and social distancing on influenza, enterovirus, and all-cause pneumonia during the coronavirus pandemic: retrospective national epidemiological surveillance study. J Med Internet Res.

[43] Chen X, Ran L, Liu Q, Hu Q, Du X, Tan X (2020). Hand hygiene, mask-wearing behaviors and its associated factors during the COVID-19 epidemic: a cross-sectional study among primary school students in Wuhan, China. Int J Environ Res Public Health.

[44] Liu JJ, Bao Y, Huang X, Shi J, Lu L (2020). Mental health considerations for children quarantined because of COVID-19. Lancet Child Adolesc Health.

[45] Huckins JF, daSilva AW, Wang W, Hedlund E, Rogers C, Nepal SK, Wu J, Obuchi M, Murphy EI, Meyer ML, Wagner DD, Holtzheimer PE, Campbell AT (2020). Mental health and behavior of college students during the early phases of the COVID-19 pandemic: longitudinal smartphone and ecological momentary assessment study. J Med Internet Res.

[46] Ye J (2020). Pediatric mental and behavioral health in the period of quarantine and social distancing With COVID-19. JMIR Pediatr Parent.

[47] Badawy SM, Radovic A (2020). Digital approaches to remote pediatric health care delivery during the COVID-19 pandemic: existing evidence and a call for further research. JMIR Pediatr Parent.

